# The impact of genetic variants in inflammatory-related genes on prostate cancer risk among men of African Descent: a case control study

**DOI:** 10.1186/1897-4287-11-19

**Published:** 2013-12-23

**Authors:** Dominique Z Jones, Camille Ragin, Nayla C Kidd, Rafael E Flores-Obando, Maria Jackson, Norma McFarlane-Anderson, Marshall Tulloch-Reid, Kevin S Kimbro, LaCreis R Kidd

**Affiliations:** 1 Department of Pharmacology & Toxicology, University of Louisville, Louisville, KY, USA; 2 Cancer Prevention and Control Program, Fox Chase Cancer Center, Philadelphia, PA, USA; 3 Molecular and Cellular Biology Program, State University of New York, Brooklyn, NY, USA; 4 Department of Community Health and Psychiatry, University of West Indies, Mona, Kingston, Jamaica; 5 Department of Basic Medical Sciences, University of the West Indies, Mona Campus, Kingston, Jamaica; 6 Tropical Medicine Research Institute, University of the West Indies, Mona, Kingston, Jamaica; 7 Biomedical/Biotechnology Research Institute (BBRI), North Carolina Central University, Durham, NC, USA

**Keywords:** Prostate cancer, Inflammatory-related sequence variants, Single nucleotide polymorphisms

## Abstract

**Purpose:**

Although case–control studies have evaluated the role of variant inflammatory-related loci in prostate cancer, their impact is virtually unknown among men of African descent. To address this, we evaluated the impact of inflammatory cytokine single nucleotide polymorphisms (SNPs) on prostate cancer risk for men of African descent.

**Methods:**

Forty-four SNPs in inflammatory cytokine-associated genes were evaluated among 814 African-American and Jamaican men (279 prostate cancer cases and 535 controls) using Illumina’s Golden gate genotyping system. Individual SNP effects were evaluated using logistic regression analysis.

**Results:**

Four SNPs were modestly associated with prostate cancer after adjusting for age. In the total population, inheritance of the *IL1R2* rs11886877 AA, *IL8RB* rs11574752 AA, *TNF* rs1800629 GA + AA, and *TNF* rs673 GA genotypes modestly increased prostate cancer risk by 1.45 to 11.7-fold relative to the referent genotype. Among U.S. men, age-adjusted dominant, recessive and additive genetic models for the *IL1R2* rs11886877 locus were linked to an increase in prostate cancer susceptibility. However, these main effects did not persist after adjusting for multiple hypothesis testing.

**Conclusion:**

Our preliminary data does not strongly support the hypothesis that inflammatory-related sequence variants influence prostate cancer risk among men of African descent. However, further evaluation is needed to assess whether other variant inflammatory-related genes may contribute to prostate cancer risk and disease progression in larger and ethnically diverse multi-center studies.

## Introduction

Chronic inflammation is thought to predispose an individual to cancer development [1]. This relationship is supported by a number of studies involving inflammatory bowel disease, colon cancer, hepatitis, liver cancer, pancreatitis, and pancreatic cancer [2-6]. Through several lines of evidence from epidemiological, histopathological, animal, genetic and molecular pathological studies, chronic inflammation is also thought to play a major role in prostate cancer development [2,3]. For example, prostatic infections have been implicated in prostate cancer either through direct or indirect promotion of the inflammatory process [1-3]. In addition, the use of non-steroidal anti-inflammatory drugs (NSAIDS) and other anti-inflammatory agents have been shown to reduce prostate cancer risk [4].

The production of cytokines can be influenced by single nucleotide polymorphisms (SNPs) detected within pro- and anti-inflammatory genes. Genetic variations in cytokine related genes may lead to alterations in the spectrum of cytokines expressed in an inflammatory environment or level of antitumor response [5]. Epidemiological studies have reported on the relationship between prostate cancer susceptibility and genes involved in the cytokine-cytokine receptor signaling pathway, such as interleukins and their receptors, ribonuclease L *(RNASEL)* and tumor necrosis factor *(TNF)*[6-13]. While men of African Descent suffer disproportionately from this disease [2,3,14,15], there is limited information about the positive link between variant cytokine genes and prostate cancer development in this population [16,17]. Therefore, additional studies are needed to investigate the role of inflammatory-related SNPs in the development of prostate cancer among individuals of African Descent.

The current study evaluated the impact of 44 inflammatory-related sequence variants in relation to prostate cancer risk among men of African Descent from the U.S. and Jamaica. Findings from our study will help to fill in the gaps in information pertinent to prostate cancer among men of African Descent.

## Materials and methods

### Study population

Our study population, 279 cases and 535 controls, was comprised of two independent case–control study sets. These studies include the Prostate Cancer Clinical Outcome Study (PC^2^OS) at the University of Louisville and the Prostate Cancer Study in Jamaica at the University of the West Indies, Mona Campus. For both study sets, all incident prostate cancer cases were histologically confirmed and the controls were assigned based on normal PSA levels, and normal DREs/biopsies. Descriptions of each contributing study have been previously described [18,19]. Briefly, the PC^2^OS study included 170 incident prostate cancer cases and 433 controls recruited between 2001–2005 through the Howard University Hospital (HUH) Division of Urology or related prostate cancer screening programs. Enrolled participants were men of African descent from the Washington, D.C. and Columbia, S.C. areas. The racial subgroups included self-reported African Americans, East African Americans, West African Americans, and Caribbean Americans. The Prostate Cancer Study in Jamaica included consecutively enrolled 109 incident prostate cancer cases and 102 controls recruited from 2005–2007 through the Urology clinic at the University Hospital of the West Indies in Kingston Jamaica.

### Criteria for inflammatory gene and SNP selection

Inflammatory-associated genes and SNPs were selected using one or more of the following criteria: (1) empirical evidence that supports a relationship between the SNP/gene and cancer or inflammatory/immune response related diseases; (2) commonly studied loci; (3) marked disparities in genotype frequency comparing men of African Descent to their Caucasian counterparts (i.e., ±10% change); (4) evidence demonstrating a link with alterations in mRNA expression/stability or protein expression/structure or function using *in silico* tools such as SNPinfo (http://snpinfo.niehs.nih.gov/snpinfo/snpfunc.htm) or published reports; and (5) a minor allele frequency ≥5% reported in the National Center for Biotechnology Information (NCBI) Entrez SNP, (http://www.ncbi.nlm.nih.gov/snp). According to NCBI, the selected SNPs had an average minor allele frequency of 22%. However, the *IL1RN* rs315951 SNP had an allele frequency of 2.1%. This rare non-synonymous sequence variant was included in the analysis to explore whether a rare SNP would lead to substantial gains in effect sizes (i.e., 2–3 fold increases in risk) and contribute to the missing genetic heritability [20,21].

### Genetic analysis of variant inflammatory-associated SNPs

Allelic discrimination of 44 inflammatory-associated sequence variants was performed using a custom Illumina GoldenGate Genotyping assay with VeraCode Technology and BeadXpress reader, according to the manufacturer’s instructions [22].

### Statistical analysis

Evaluation of the relationship between variant inflammatory associated alleles and prostate cancer risk was performed using univariate and multivariate analyses. The chi-square test of heterogeneity was used to assess for significant differences in the distribution of homozygous major, heterozygous, or homozygous minor genotypes between prostate cancer cases and controls. Evaluation of the relationship between prostate cancer risk and selected polymorphic genes, expressed as odds ratios (ORs) and corresponding 95% confidence intervals (CIs), were estimated using unconditional multivariate LR models adjusted for age. The major or common genotype was used as the reference category for each LR model. Statistical significance was assessed using a Bonferroni Correction (α = 0.05/44 SNPs) cut-off of 0.001, in order to adjust for multiple comparisons. All statistical analyses were performed using SAS 9.3 (SAS Institute Inc., Cary, NC) and SNP Variation Software 7.0 (Golden Helix, Bozeman, MT).

### Statistical power

Based on our sample size for the total population, U.S. and Jamaican men, we had >80% power to detect SNPs with odds ratios (ORs) of ≥1.4, ≥1.6, ≥1.8, respectively, for a co-dominant genetic model with 1 degree of freedom (df), a minor allele frequency of at least 22% and disease prevalence of 0.74%. Analyses were performed using Power for Genetic Association Version 2 Software [23].

## Results

### Prevalence of inflammatory-associated sequence variants among men of African Descent

Inheritance of variant inflammatory-related loci was fairly common among African-American men in the current study. Specifically, the minor allele frequencies of the 44 sequence variants ranged from approximately 2.6% to 48%, as depicted in Table 1. Notably, the observed genotype frequency distribution among controls did not significantly deviate from expected counts according to the Hardy Weinberg equilibrium. With the exception of four loci (*IL1RN* rs4251961, *IL10RB* rs999788, IL10RB rs283416, and ILR1 rs3917225), the observed genotype frequencies in the current study corroborated with values for individuals of African-American/African ancestry reported in the NCBI’s SNP entrez (P = 0.063-1.000), as shown in Table 1.

**Table 1:** Functional consequence and prevalence of inflammatory-associated sequence variants

**dbSNPID**	**Gene**	**Location functional consequence**	**NCBI nucleotide change (major > minor allele)**^**†**^	**NCBI minor allele frequency (MAF) for African-Americans**	**NCBI major/major genotype n (%) for African-Americans**	**NCBI major/minor genotype n (%) for African-Americans**	**NCBI minor/minor genotype n (%) for African-Americans**	**Current study nucleotide change (major > minor allele)**	**Current study MAF n(%) for African-Americans**	**Current study major/major genotype n (%) for African-Americans**	**Current study major/minor genotype n (%) for African-Americans**	**Current study minor/minor genotype n (%) for African-Americans**	**Overall χ**^**2 **^**P-value comparing genotypes from individuals of African Descent as reported in NCBI versus the current study**^**††**^
**rs1058867**^**ǂ**^	IL10RB	UTR'3 miRNA	G > A	A = 37.9	26 (42.0)	25 (40.3)	11 (17.7)	G > A	A = 33.9	239 (44.6)	229 (42.9)	67 (12.5)	0.514
**rs1071676**^**ǂ**^	IL1B	UTR'3 miRNA	G > C	C = 14.6	19 (79.2)	3 (12.5)	2 (8.3)	G > C	C = 16.1	378 (70.7)	142 (26.5)	15 (2.80)	*0.106*
**rs11123902**^**ǂ**^	IL1R2	Intron 1	A > C	C = 31.8	10 (45.5)	10 (45.5)	2 (9.10)	A > C	C = 30.7	258 (48.2)	225 (42.1)	52 (9.70)	*0.949*
**rs1126579**^**ǂ**^	IL8RB	UTR'3 miRNA	C > T	T = 14.5	46 (74.2)	14 (22.6)	2 (3.20)	G > A^**†**^	A = 13.8	402 (75.1)	118 (22.1)	15 (2.80)	*0.886*
**rs1143627**^**ǂ**^	IL1B	Near gene 5' TFBS	C > T	T = 37.5	12 (50.0)	6 (25.0)	6 (25.0)	G > A^**†**^	A = 39.6	194 (36.3)	256 (48.2)	83 (15.5)	*0.063*
**rs1143634**^**ǂ**^	IL1B	Exon 4 Splicing	C > T	T = 12.9	47 (75.8)	14 (22.6)	1 (1.60)	G > A^**†**^	A = 15.5	381 (71.2)	142 (26.5)	12 (2.3)	*0.833*
**rs11574752**^**ǂ**^	IL8RB	UTR'3 miRNA	G > A	A = 10.4	19 (79.2)	5 (20.8)	0(0.00)	G > A	A = 9.40	435 (81.3)	99 (18.5)	1 (0.20)	*0.798*
**rs11886877**	IL1R2	Intron 1	---	---	---	---	---	G > A	A = 35.8	211 (39.4)	265 (49.5)	59 (11.1)	
**rs12135247**^**ǂǂ**^	RNASEL	UTR'3 TFBS, miRNA	T > C	C = 16.3	33 (67.3)	16 (32.7)	0 (0.00)	A > G^**†**^	G = 17.9	368 (68.8)	142 (26.5)	25 (4.70)	*0.226*
**rs12328606**^**ǂǂ**^	IL1R2	Near gene 5' TFBS	C > T	T = 11.2	38 (77.6)	11 (22.4)	0 (0.00)	G > A^**†**^	A = 13.5	405 (75.7)	116 (21.7)	14 (2.60)	*0.798*
**rs1304037**^**ǂ**^	IL1A	UTR'3 miRNA	A > G	G = 39.6	8 (33.3)	13 (54.2)	3 (12.5)	A > G	G = 41.1	191 (35.7)	248 (46.4)	96 (17.9)	*0.760*
**rs16944**^**ǂ**^	IL1B	Near gene 5' TFBS	A > G	G = 39.0	18 (30.5)	36 (61.0)	5 (8.50)	A > G	G = 45.1	156 (29.2)	275 (51.4)	104 (19.4)	0.108
**rs17561**^**ǂ**^	IL1A	Exon 4 Splicing, nsSNP, benign	G > T	T = 15.3	44 (71.0)	17 (27.4)	1 (1.60)	C > A^**†**^	A = 18.6	358 (66.9)	155 (29.0)	22 (4.10)	*0.698*
**rs1799964**^**ǂ**^	TNF	Near gene 5' TFBS	T > C	C = 12.9	46 (74.2)	16 (25.8)	0 (0.00)	A > G^**†**^	G = 16.5	374 (69.9)	145 (27.1)	16 (3.00)	*0.492*
**rs1800587**^**ǂ**^	IL1A	UTR'5 TFBS, Splicing	C > T	T = 39.1	9 (39.1)	10 (43.5)	4 (17.4)	G > A^**†**^	A = 41.8	183 (34.2)	257 (48.0)	95 (17.8)	*0.885*
**rs1800629**^**ǂ**^	TNF	Near gene 5' TFBS	G > A	A = 13.7	46 (74.2)	15 (24.2)	1 (1.60)	G > A	A = 16.9	368 (68.8)	153 (28.6)	14 (2.60)	0.752
**rs1800871**^**ǂ**^	IL10	Near gene 5' TFBS	C > T	T = 36.3	28 (45.2)	23 (37.1)	11 (17.7)	G > A^**†**^	A = 40.7	188 (35.0)	258 (48.0)	89 (17.0)	0.219
**rs1800872**^**ǂǂ**^	IL10	Near gene 5' TFBS	A > C	A = 50.0 C = 50.0	5 (21.7)	13 (56.6)	5 (21.7)	C > A^**†**^	A = 40.7	188 (35.0)	258 (48.0)	89 (17.0)	0.874
**rs1800893**^**ǂ**^	IL10	Near gene 5' TFBS	G > A	A = 37.1	22 (35.5)	34 (54.8)	6 (9.70)	G > A	A = 36.4	216 (40.4)	248 (46.4)	71 (13.2)	0.419
**rs1800896**^**ǂ**^	IL10	Near gene 5' TFBS	A > G	G = 40.5	7 (33.3)	11 (52.4)	3 (14.3)	A > G	G = 33.3	243 (45.4)	228 (42.6)	64 (12.0)	*0.491*
**rs2192752**^**ǂ**^	IL1R1	Near gene 5' TFBS	A > C	C = 4.80	56 (90.3)	6 (9.70)	0 (0.00)	A > C	C = 5.60	477 (89.1)	56 (10.5)	2 (0.40)	*1.000*
**rs2227532**^**ǂ**^	IL8	Near gene 5' TFBS	T > C	C = 9.70	50 (80.6)	12 (19.4)	0 (0.00)	A > G^**†**^	G = 8.60	448 (83.7)	82 (15.3)	5 (1.00)	*0.690*
**rs2227538**^**ǂ**^	IL8	UTR'5 TFBS, Splicing	C > T	T = 17.7	41 (66.1)	20 (32.3)	1 (1.60)	G > A^**†**^	A = 23.2	323 (60.4)	176 (32.9)	36 (6.70)	*0.291*
**rs2227545**^**ǂ**^	IL8	UTR'3 miRNA	A > C	C = 8.70	19 (82.6)	4 (17.4)	0 (0.00)	A > C	C = 8.50	449 (83.9)	81 (15.1)	5 (1.00)	*0.812*
**rs2229113**^**ǂ**^	IL10RA	Exon 7 nsSNP, probably damaging	G > A	A = 20.5	14 (63.7)	7 (31.8)	1 (4.50)	G > A	A = 18.8	355 (66.4)	159 (29.7)	21 (3.90)	*0.782*
**rs2834167**^ǂ^	IL10RB	Exon 2 Splicing, nsSNP, benign	A > G	G = 16.9	44 (71.0)	15 (24.2)	3 (4.80)	A > G	G = 11.0	423 (79.1)	106 (19.8)	6 (1.10)	*0.050*
**rs2856836**^**ǂ**^	IL1A	UTR'3 miRNA	T > C	C = 17.4	16 (69.6)	6 (26.1)	1 (4.30)	A > G^**†**^	G = 18.6	358 (66.9)	155 (29.0)	22 (4.10)	*1.000*
**rs3135932**^**ǂ**^	IL10RA	Exon 5 Splicing, nsSNP, benign	A > G	G = 2.10	23 (95.8)	1 (4.20)	0 (0.00)	A > G	G = 2.60	508 (95.0)	26 (4.80)	1 (0.20)	*1.000*
**rs315951**^**ǂ**^	IL1RN	UTR'3 miRNA	C > G	G = 47.9	8 (33.3)	9 (37.5)	7 (29.2)	C > G	G = 48.0	144 (26.9)	268 (50.1)	123 (23.0)	0.482
**rs3738579**^**ǂ**^	RNASEL	UTR'5 TFBS, Splicing	T > C	C = 16.7	14 (66.7)	7 (33.3)	0 (0.00)	A > G^**†**^	G = 12.3	411 (76.8)	116 (21.7)	8 (1.50)	*0.474*
**rs3917225**^**ǂ**^	IL1R1	Near gene 5' TFBS	A > G	G = 12.3	49 (80.3)	9 (14.8)	3 (4.90)	A > G	G = 9.10	438 (81.9)	97 (18.1)	0 (0.00)	*0.001*
**rs4073**^**ǂ**^	IL8	Near gene 5' TFBS	A > T	T = 26.6	34 (54.8)	23 (37.1)	5 (8.10)	T > A	A = 20.9	335 (62.6)	176 (32.9)	24 (4.50)	*0.262*
**rs4141134**^**ǂǂ**^	IL1R2	Near gene 5'	T > C	C = 11.2	39 (79.6)	9 (18.4)	1 (2.00)	A > G^**†**^	G = 13.7	399 (74.6)	125 (23.4)	11 (2.00)	*0.660*
**rs4251961**^**ǂ**^	IL1RN	Near gene 5' TFBS	T > C	C = 20.2	37 (59.7)	25 (40.3)	0 (0.00)	A > G^**†**^	G = 17.9	367 (68.6)	145 (27.1)	23 (4.30)	*0.035*
**rs4252243**^**ǂ**^	IL10RA	Near gene 5' TFBS	C > T	T = 32.5	9 (45.0)	9 (45.0)	2 (10.0)	G > A^**†**^	A = 27.5	271 (50.7)	234 (43.7)	30 (5.60)	*0.522*
**rs4674257**^**ǂǂ**^	IL8RB	Near gene 5' TFBS	G > A	A = 25.0	14 (58.3)	8 (33.3)	2 (8.30)	G > A	A = 20.1	347 (64.9)	161 (30.10)	27 (5.00)	*0.468*
**rs4674259**^**ǂ**^	IL8RB	UTR'5 TFBS	A > G	G = 23.9	12 (52.2)	11 (47.8)	0 (0.00)	A > G	G = 20.0	349 (65.0)	158 (30.0)	28 (5.00)	*0.176*
**rs486907**^**ǂ**^	RNASEL	Exon 1 nsSNP, benign	G > A	A = 16.7	16 (66.7)	8 (33.3)	0 (0.00)	G > A	A = 13.2	402 (75.1)	125 (23.4)	8 (1.50)	*0.528*
**rs6726713**^**ǂǂ**^	IL1R2	Near gene 5' TFBS	C > T	T = 11.2	38 (77.6)	11 (22.4)	0 (0.00)	G > A^**†**^	A = 12.1	417 (78.0)	106 (19.8)	12 (2.20)	*0.689*
**rs673**^**ǂ**^	TNF	Near gene 5' TFBS	G > A	A = 13.7	45 (72.6)	17 (27.4)	0 (0.00)	G > A	A = 17.4	364 (68.0)	156 (29.2)	15 (2.80)	*0.546*
**rs8178433**^**ǂ**^	IL10RB	Near gene 5' TFBS	T > G	G = 12.9	46 (74.2)	16 (25.8)	0 (0.00)	A > C^**†**^	C = 12.4	408 (76.3)	121 (22.6)	6 (1.10)	*0.811*
**rs949963**^**ǂ**^	IL1R1	Near gene 5' TFBS	G > A	A = 31.1	31 (50.8)	22 (36.1)	8 (13.1)	G > A	A = 33.1	249 (46.5)	218 (40.8)	68 (12.7)	0.771
**rs9610**^**ǂ**^	IL10RA	UTR'3 miRNA	A > G	G = 41.9	20 (32.3)	32 (51.6)	10 (16.1)	A > G	G = 33.9	237 (44.3)	233 (43.5)	65 (12.2)	0.184
**rs999788**^**ǂ**^	IL10RB	Near gene 5' TFBS	C > T	T = 19.5	40 (67.8)	15 (25.4)	4 (6.80)	G > A^**†**^	A = 12.4	410 (76.6)	117 (21.9)	8 (1.50)	*0.026*

^**†**^The nucleotide change may vary relative to that reported in NCBI depending on whether the genotyping was performed using the sense or anti-sense DNA strand.

^**††**^The chi-square test was used to assess differences in the overall genotype frequencies comparing men of African Descent as reported in NCBI to those in the total population from the current study**.** P-values generated from the Fisher’s exact test (in italics) were used when expected genotype counts were < 5 for either cases or controls.

Abbreviations: *MAF* Minor Allele Frequency; *UTR* untranslated region; *TFBS* transcription factor binding site; *nsSNP* non-synonymous coding SNP; *miRNA* microRNA binding site; *NCBI* National Center for Biotechnology Information Entrez SNP.

^ǂ^NCBI AFR1 or African American Population Panel.

^ǂǂ^NCBI ASW Population Panel.

### Relationship between inflammatory sequence variants and prostate cancer risk

Seven out of 44 sequence variants detected in inflammatory-related genes were modestly associated with prostate cancer risk among 814 men of African Descent (279 cases and 535 controls), as summarized in Table 2. For age-adjusted risk models, elevations in prostate cancer susceptibility were observed among carriers of *IL1R2* rs1188687 7AA (OR = 1.92; 95%CI = 1.11, 3.32), *IL8RB* rs11574752 GA + AA (OR = 38.40; 95%CI = 3.86, 382.8), *TNF* rs1800629 GA + AA (OR = 1.53; 95%CI = 1.06, 2.20), and *TNF* rs673 GA (OR = 1.50; 95%CI = 1.04, 2.16) genotypes with risk estimates ranging from 1.50-38.4. The *IL1R2* rs11886877 marker was the only genetic susceptibility factor significant under the additive genetic model (P-trend = 0.010), indicative of a significant dose–response effect in relation to the number of inherited minor alleles. The aforementioned markers were not classified as important prostate cancer risk indicators after adjusting for multiple comparisons bias using the Bonferroni correction, with a significance cut-off of ≤0.001.

**Table 2:** Relationship between inflammatory related sequence variants and prostate cancer risk among men of African Descent

**Genes**	**dbSNP ID location predicted function**	**Genotype**	**Cases n (%)**	**Controls n (%)**	**Unadjusted OR (95%CI)**^**†**^	**Adjusted OR (95%CI)**^**†**^	**p-value**^**ǂ**^	**p trend**	**Bonferroni correction**
*IL1R2*	rs11886877	GG	87 (31.2)	211 (39.4)	1.00 (referent)	1.00 (referent)	**0.034**	**0.010**	NS
	Intron 1	GA	149 (53.4)	265 (49.5)	1.36 (0.99, 1.88)	1.35 (0.92,1.98)	0.058		
		AA	43 (15.4)	59 (11.1)	**1.77 (1.11, 2.82)**	**1.92 (1.11, 3.32)**	**0.017**		
		GA + AA	192 (68.8)	324 (60.6)	**1.44 (1.06, 1.95)**	**1.46 (1.01, 2.10)**	**0.021**		
		AA vs (GG + GA)			1.47 (0.96,2.24)	1.61 (0.98,2.63)	0.074		
*IL1A*	rs17561	CC	195 (69.9)	358 (66.9)	1.00 (referent)	1.00 (referent)	**0.025**	0.108	NS
	Exon 4	CA	82 (29.4)	155 (29.0)	0.97 (0.70,1.34)	1.01 (0.68,1.48)	0.858		
	Splicing	AA	2 (0.70)	22 (4.10)	**0.17 (0.04, 0.72)**	0.40 (0.08,1.83)	**0.016**		
	nsSNP	CA + AA	84 (30.1)	177 (33.1)	0.87 (0.64,1.20)	0.96 (0.66,1.40)	0.388		
	benign	AA vs (CC + CA)			**0.17 (0.04, 0.72)**	0.40 (0.09,1.82)	**0.016**		
*IL8RB*	rs11574752	GG	230 (82.4)	435 (81.3)	1.00 (referent)	1.00 (referent)	***0.011***	0.784	NS
	3′-UTR	GA	43 (15.4)	99 (18.5)	0.82 (0.55,1.21)	0.90 (0.56,1.40)	0.326		
	miRNA	AA	6 (2.20)	1 (0.20)	**11.3 (1.36, 94.6)**	**38.4 (3.86, 382.8)**	***0.009***		
		GA + AA	49 (17.6)	100 (18.7)	0.93 (0.64,1.35)	1.08 (0.69,1.70)	0.693		
		AA vs (GG + GA)			**11.7 (1.40, 98.0)**	**39.2 (3.94, 390)**	***0.008***		
*TNF*	rs1800629	GG	171 (61.2)	368 (68.8)	1.00 (referent)	1.00 (referent)	**0.047**	0.087	NS
	5′ near gene	GA	103 (37.0)	153 (28.6)	**1.45 (1.06, 1.97)**	**1.54 (1.06, 2.24)**	**0.019**		
	TFBS	AA	5 (1.80)	14 (2.60)	0.77 (0.27, 2.16)	1.30 (0.37,4.60)	0.619		
		GA + AA	108 (38.8)	167 (31.2)	**1.39 (1.03, 1.90)**	**1.53 (1.06, 2.20)**	**0.032**		
		AA vs (GG + GA)			0.68 (0.24,1.91)	1.13 (0.32,3.90)	0.462		
*TNF*	rs673	GG	171 (61.3)	364 (68.0)	1.00 (referent)	1.00 (referent)	**0.009**	0.228	NS
	5′ near gene	GA	106 (38.0)	156 (29.2)	**1.45 (1.06, 2.00)**	**1.50 (1.04, 2.16)**	**0.018**		
	TFBS	AA	2 (0.70)	15 (2.80)	0.28 (0.06, 1.26)	0.47 (0.09,2.40)	0.097		
		GA + AA	108 (39.1)	171 (32.0)	1.34 (0.99, 1.82)	1.43 (1.00, 2.05)	0.055		
		AA vs (GG + AG)			0.25 (0.06,1.10)	0.41 (0.08,2.07)	0.067		
*IL1A*	rs2856836	AA	196 (70.3)	358 (66.9)	1.00 (referent)	1.00 (referent)	**0.024**	0.089	NS
	3′-UTR	AG	81 (29.0)	155 (29.0)	0.96 (0.69,1.32)	0.99 (0.67,1.45)	0.776		
	miRNA	GG	2 (0.70)	22 (4.10)	**0.17 (0.04, 0.71)**	0.40 (0.09,1.82)	**0.016**		
		AG + GG	83 (29.7)	177 (33.1)	0.86 (0.63,1.17)	0.94 (0.65,1.36)	0.333		
		GG vs (AA + AG)			**0.17 (0.04, 0.72)**	0.40 (0.09,1.82)	**0.016**		
*IL10RA*	rs4252243	GG	134 (48.4)	268 (50.4)	1.00 (referent)	1.00 (referent)	0.066	0.168	NS
	5′ near gene	GA	115 (41.5)	234 (44.0)	0.98 (0.72,1.32)	0.83 (0.58,1.18)	0.893		
	TFBS	AA	28 (10.1)	30 (5.60)	**1.86 (1.07, 3.24)**	1.62 (0.82, 3.21)	**0.028**		
		GA + AA	143 (51.6)	264 (49.6)	1.08 (0.81,1.44)	0.91 (0.64,1.28)	0.605		
		AA vs (GG + GA)			**1.88 (1.10, 3.21)**	1.77 (0.91, 3.43)	**0.021**		

^ǂ^On a separate line before the text regarding the chi-square test p-values state the following:

^†^Boldface odd ratios (ORs) and 95% confidence interval (CI) indicate a significant relationship between the selected SNPs and prostate cancer risk.

From top to bottom within the column, the chi-square test p-values were used to determine the difference in the genotype frequencies between cases and controls for the overall, minor/major versus major/major genotypes, as well as the dominant (i.e., minor/minor versus major/major), co-dominant (minor/minor + major/minor versus major/major), and recessive genetic models (minor/minor versus major/major + major/minor). P-values generated from the Fisher’s Exact test (in italics) were calculated when expected genotype counts were < 5 for either cases or controls. Statistically significant p-values are marked in bold face.

Abbreviations: *UTR,* untranslated region; *TFBS,* transcription factor binding site; *miRNA,* microRNA binding site; *NS,* non-significant.

Upon stratification by sub-population, modestly significant prostate cancer biomarkers varied by racial/ethnic group in the age adjusted risk models. Possession of the *RNASEL* rs1213524 AG genotype was associated with a 2.17-fold increase in the risk of developing prostate cancer (OR = 2.10; 95%CI = 1.04, 4.24) among Jamaican men, as detailed in Table 3. However, this locus was not significant in the dominant, recessive or additive genetic models. Similar to the total population, inheritance of sequence variants in *IL1R2*, *IL10RA* and *TNF* among U.S. men were linked with a significant increase in prostate cancer risk. Among U.S. men, two inflammatory-related sequence variants, [*IL1R2* rs11886877 (GA, GA + AA, AA) and *IL10RA* rs4252243 AA], were associated with a 1.82-2.49-fold increase in prostate cancer risk. Out of these 2 markers, the *IL1R2* rs11886877 locus was significant for the dominant (OR = 2.75; 95%CI = 1.38, 5.50), co-dominant (OR = 1.82; 95%CI = 1.14, 2.88), recessive (OR = 2.05; 95%CI = 1.10, 3.80), and additive (P-trend value = 0.002) genetic models. None of the aforementioned markers survived correction for multiple hypotheses testing. Moreover, the IL10RA rs4252243 SNP was only significantly related to prostate cancer risk under the recessive genetic model (OR = 2.49; 95%CI = 1.08, 5.72).

**Table 3:** Relationship between inflammatory related sequence variants and prostate cancer risk among U.S. and Jamaican men

**Genes**	**dbSNP ID location predicted function**	**Genotype**	**Unadjusted OR (95%CI) US men†**	**Age-adjusted OR (95%CI) US men†**	**Unadjusted OR (95%CI) Jamaican men†**	**Age-adjusted OR (95%CI) Jamaican men†**	**p-value US men**^**ǂ**^	**p-value Jamaican men**^**ǂ**^	**p-trend US men**	**p-trend Jamaican men**
*IL1B*	rs1071676	GG	1.00 (referent)	1.00 (referent)	1.00 (referent)	1.00 (referent)	***0.050***	*0.550*	**0.022**	0.276
	UTR'3	GC	0.72 (0.48, 1.10)	0.70 (0.42, 1.14)	1.39 (0.71, 2.70)	1.28 (0.62, 2.64)	0.124	0.338		
	miRNA	CC	0.16 (0.02, 1.25)	0.19 (0.02, 2.00)	2.02 (0.18, 22.8)	1.15 (0.10, 14.6)	***0.035***	*0.500*		
		GC + CC	0.66 (0.44, 1.00)	0.66 (0.40, 1.10)	1.42 (0.74, 2.72)	1.26 (0.62, 2.60)	**0.050**	0.294		
		CC vs (GG + GC)	0.18 (0.02, 1.36)	0.21 (0.02, 2.18)	1.89 (0.16, 21.1)	1.10 (0.08, 13.7)	***0.046***	*0.525*		
*IL1B*	rs1143634	GG	1.00 (referent)	1.00 (referent)	1.00 (referent)	1.00 (referent)	*0.051*	*0.447*	**0.016**	0.203
	Exon 4	GA	0.67 (0.44, 1.01)	0.65 (0.40, 1.06)	1.51 (0.76, 3.00)	1.37 (0.64, 2.90)	0.058	0.243		
	Splicing	AA	0.21 (0.02, 1.60)	0.24 (0.02, 2.86)	2.05 (0.18, 23.0)	1.16 (0.10, 14.6)	*0.080*	*0.496*		
		GA + AA	**0.63 (0.42, 0.95)**	0.62 (0.38, 1.02)	1.54 (0.78, 3.00)	1.36 (0.65, 2.82)	**0.028**	0.208		
	cds-synonymous	AA vs (GG + GA)	0.23 (0.02, 1.80)	0.27 (0.02, 3.20)	1.89 (0.16, 21.1)	1.10 (0.08, 13.7)	*0.105*	*0.525*		
*IL1R2*	rs11886877	GG	1.00 (referent)	1.00 (referent)	1.00 (referent)	1.00 (referent)	**0.007**	0.889	**0.002**	0.631
	Intron 1	GA	**1.60 (1.08, 2.40)**	1.63 (1.00, 2.64)	0.92 (0.50, 1.68)	0.94 (0.48, 1.80)	**0.020**	0.782		
		AA	**2.34 (1.31, 4.16)**	**2.75 (1.38, 5.50)**	0.82 (0.36, 1.86)	0.94 (0.38, 2.30)	**0.004**	0.633		
		GA + AA	**1.72 (1.18, 2.52)**	**1.82 (1.14, 2.88)**	0.89 (0.50, 1.58)	0.94 (0.50, 1.74)	**0.005**	0.700		
		AA vs (GG + GA)	**1.76 (1.04, 2.96)**	**2.05 (1.10, 3.80)**	0.86 (0.40, 1.80)	0.97 (0.43, 2.20)	**0.033**	0.691		
*RNASEL*	rs12135247	AA	1.00 (referent)	1.00 (referent)	1.00 (referent)	1.00 (referent)	0.800	***0.025***	0.909	0.216
	UTR'3	AG	1.06 (0.72, 1.58)	1.14 (0.71, 1.84)	**2.17 (1.14, 4.12)**	**2.10 (1.04, 4.24)**	0.756	**0.018**		
	TFBS	GG	0.77 (0.30, 1.96)	0.70 (0.24, 2.10)	0.45 (0.08, 2.40)	0.28 (0.04, 1.70)	0.570	*0.284*		
	miRNA	AG + GG	1.02 (0.70, 1.50)	1.07 (0.68, 1.68)	1.81 (0.99, 3.30)	1.68 (0.88, 3.24)	0.906	0.053		
		GG vs (AG + AA)	0.76 (0.30, 1.92)	0.67 (0.22, 1.97)	0.36 (0.06, 1.91)	0.22 (0.04, 1.35)	0.555	*0.196*		
*TNF*	rs1800629	GG	1.00 (referent)	1.00 (referent)	1.00 (referent)	1.00 (referent)	*0.101*	*0.782*	0.113	0.549
	5′ near gene	GA	**1.50 (1.03, 2.20)**	1.52 (0.96, 2.42)	1.21 (0.68, 2.12)	1.41 (0.78, 2.63)	**0.034**	0.518		
	TFBS	AA	0.90 (0.28, 2.80)	1.51 (0.36, 6.24)	1.00 (0.06, 16.3)	1.00 (0.06, 17.2)	*0.551*	*0.752*		
		GA + AA	1.44 (0.99, 2.10)	1.53 (0.97, 2.40)	1.20 (0.68, 2.10)	1.40 (0.75, 2.60)	**0.050**	0.525		
		AA vs (GG + GA)	0.78 (0.25, 2.42)	1.32 (0.32, 5.40)	0.94 (0.06, 15.2)	0.88 (0.05, 15.0)	*0.452*	*0.734*		
*IL1A*	rs1800587	GG	1.00 (referent)	1.00 (referent)	1.00 (referent)	1.00 (referent)	0.088	0.450	**0.028**	0.224
	UTR'5	GA	0.75 (0.50, 1.10)	0.68 (0.42, 1.08)	1.38 (0.76, 2.50)	1.42 (0.74, 2.72)	0.144	0.297		
	TFBS	AA	**0.56 (0.32, 0.96)**	0.78 (0.40, 1.53)	1.56 (0.69, 3.50)	1.64 (0.70, 4.00)	**0.038**	0.279		
	Splicing (ESE or	GA + AA	0.70 (0.48, 1.00)	0.70 (0.45, 1.10)	1.42 (0.80, 2.50)	1.47 (0.80, 2.72)	0.053	0.222		
	ESS)	AA vs (GG + GA)	0.66 (0.40, 1.10)	0.96 (0.52, 1.80)	1.30 (0.62, 2.70)	1.34 (0.60, 3.00)	0.105	0.478		
*IL10RA*	rs4252243	GG	1.00 (referent)	1.00 (referent)	1.00 (referent)	1.00 (referent)	0.062	0.620	0.275	0.329
	5′ near gene	GA	0.92 (0.63, 1.33)	0.70 (0.44, 1.10)	1.24 (0.70, 2.20)	1.21 (0.64, 2.28)	0.648	0.448		
	TFBS	AA	**2.02 (1.04, 3.95)**	2.10 (0.90, 4.98)	1.50 (0.54, 4.26)	1.04 (0.34, 3.20)	**0.038**	0.436		
		GA + AA	1.03 (0.72, 1.50)	0.81 (0.52, 1.30)	1.28 (0.74, 2.21)	1.15 (0.64, 2.10)	0.863	0.328		
		AA vs (GG + GA)	**2.11 (1.10, 4.02)**	**2.49 (1.08, 5.72)**	1.37 (0.50, 3.74)	1.02 (0.40, 2.98)	**0.023**	0.539		
*TNF*	rs673	GG	1.00 (referent)	1.00 (referent)	1.00 (referent)	1.00 (referent)	***0.027***	*0.452*	0.279	0.874
	5′ near gene	GA	**1.50 (1.02, 2.20)**	1.46 (0.92, 2.30)	1.22 (0.70, 2.14)	1.41 (0.76, 2.62)	**0.025**	0.498		
	TFBS	AA	0.24 (0.03, 1.84)	0.54 (0.06, 4.44)	0.33 (0.03, 3.24)	0.40 (0.03, 4.70)	*0.116*	*0.315*		
		GA + AA	1.38 (0.95, 2.00)	1.40 (0.88, 2.20)	1.14 (0.66, 2.00)	1.33 (0.72, 2.46)	0.087	0.635		
		AA vs (GG + GA)	0.21 (0.02, 1.60)	0.47 (0.06, 3.91)	0.31 (0.03, 2.98)	0.35 (0.03, 4.08)	*0.080*	*0.286*		

On a separate line before the text regarding the chi-square test p-values state the following:

†Boldface odd ratios (ORs) and 95% confidence interval (CI) indicate a significant relationship between the selected SNPs and prostate cancer risk.

^ǂ^From top to bottom within the column, the chi-square test p-values were used to determine the difference in the genotype frequencies between cases and controls for the overall, minor/major versus major/major genotypes, as well as the dominant (i.e., minor/minor versus major/major), co-dominant (minor/minor + major/minor versus major/major), and recessive genetic models (minor/minor versus major/major + major/minor). P-values generated from the Fisher’s Exact test (in italics) were calculated when expected genotype counts were < 5 for either cases or controls. Statistically significant p-values are marked in bold face.

Abbreviations: *UTR,* untranslated region; *TFBS,* transcription factor binding site; cds-syn, synonymous SNP; *miRNA,* microRNA binding site.

## Discussion

Chronic inflammation has been associated with tumor development and metastasis. Inflammatory response is regulated through a complex network of cytokines, cytokine receptors and downstream targets that synergistically regulate innate/humoral immune and inflammatory processes. Recent molecular and genetic epidemiology studies have demonstrated that chronic inflammation and susceptibilities in inflammatory-associated genes are related to the development of several cancers, including lymphoma, and gastric and prostate cancer [6,16,24-26]. However, to our knowledge, there are few published reports on the impact of variant cytokine-related genes in relation to prostate cancer among men of African Descent. Therefore, the current study evaluated the individual effects of 44 inflammatory associated sequence variants on prostate cancer risk among 279 cases and 535 disease-free men of African Descent from the U.S and Jamaica. Our findings revealed a modest increase in prostate cancer risk for unadjusted and adjusted logistic regression models for *IL1R2* rs11886877 among men of African Descent. The additive, dominant and recessive genetic models of this variant were significant even after adjusting for age. However, this relationship did not survive after accounting for multiple comparisons bias.

*IL1R2* rs11886877 is about 2415 base pairs from the transcription start site, which suggest it may have a high likelihood of regulating *IL1R2* gene expression. Currently, there are no published reports on the relationship between *IL1R2* rs11886877 and prostate cancer for any population. Although there is no evidence of the impact of this sequence variant on prostate cancer risk among European and African American men, the relationship between the *IL1R2* gene expression and prostate cancer has been demonstrated through published reports [27-29]. Leshem and colleagues (2011) found that the promoter region of *IL1R2* possesses putative binding motifs for the *TMPRSS2/ERG* fusion gene, which is highly expressed in aggressive prostate cancer [27]. When the expression of *IL1R2* was knocked down using small interfering RNAs, it resulted in the reduction of *ZEB2* mRNA expression in hTERT/shp53/CyclinD-CDK4 overexpressing cells exposed to *TMPRSS2/ERG*[25]. *TMPRSS2/ERG* fusion gene indirectly up-regulates ZEB2, a facilitator of the epithelial to mesenchymal transition (EMT), by binding to IL1R2 to increase prostate cancer tumorigenesis [30].

Out of 44 inflammatory-related sequence variants, 7 SNPs included in our study were evaluated in relation to prostate cancer outcomes within 4 independent observational studies [6,7,10,11]. Commensurate with our findings, two observational studies demonstrated that sequence variants detected in *IL10* (rs1800871, rs1800872) and *IL8* rs4073 were not significantly related to prostate cancer risk [6,11]. Inheritance of the *TNF* rs1800629 AA genotype was associated with a significant 1.8 fold increase in prostate cancer risk among Caucasian men in a small study (150 cases, 150 controls); however, this marker resulted in null findings in a larger study (468 cases, 468 controls) [6,7]. In our preliminary analyses, inheritance of one or more *TNF* rs1800629 A alleles was marginally associated with a 1.5-fold increase in prostate risk; however, this relationship did not survive adjustment after multiple hypothesis testing. Lastly, *IL10* rs1800896 G and *IL1B* rs1143627 C alleles had protective effects in two separate Caucasian sub-populations. However, neither of these markers were significantly related to prostate cancer among African-American men in the current study. Casey and colleagues (2002) showed a 2-fold increase in prostate cancer susceptibility linked to inheritance of the *RNASEL* rs486907 AA genotype among mostly men of European descent [10]. This locus was not related to prostate cancer risk among African-Americans in the current study. Racial/ethnic disparities in the aforementioned risk estimates may be attributed to differences in minor allele frequencies, failure to adjust findings for multiple hypothesis testing or inadequate sample size among men with African ancestry.

In this study, we considered the strengths, limitations and future directions of the project. Forty-four sequence variants were evaluated in relation to prostate cancer risk among men of African Descent from the U.S. and Jamaica. Upon stratification by study center, the *IL1R2* rs11886877 locus was marginally related to prostate cancer among men of African descent from the U.S. However, overall the inflammatory-related sequence variants were not robustly related to prostate cancer among our study participants. Despite this, we cannot eliminate the possibility that *IL1R2* and other inflammatory-related sequence variants not included in this study may influence the risk of prostate cancer development or aggressive tumor behavior. In larger studies, the impact of individual or interaction among inflammatory cytokine-associated sequence variants in relation to prostate cancer tumor grade, biochemical or disease recurrence, and mortality using targeted sequencing, *in vitro* studies, *in silico* and bioinformatics tools. Such efforts will help to identify genetic markers linked to disproportionately high prostate cancer incidence, mortality, and morbidity rates among men of African Descent. Population admixture, which commonly occurs among men of African descent, may bias risk estimates. However, adjustment of risk estimates by West African Ancestry and/or age did not significantly modify the directionality of observed risk estimates among men from the U.S. (data not shown). Although, the sample size of this study population is small, there was ample statistical power to accurately detect risk estimates, ranging between 1.4-1.8 or 0.55-0.70. Our findings are important to genetic epidemiology research teams interested in pooling genetic and tumor characteristic data to determine whether other variant inflammatory-related cytokines contribute to prostate cancer susceptibility and disease prognosis. Although this study displays a modest association between inflammatory-related cytokine variant *IL1R2* rs11886877 and prostate cancer risk, this relationship has yet to be tested biologically. The association of *IL1R2* rs11886877 with prostate cancer risk may prove to be strong in a larger study population.

## Conclusions

Chronic inflammation is an established risk factor of prostate cancer and many studies argue that it can lead to prostate cancer development. In this study, 44 inflammatory-related cytokine variants that may play a role in chronic inflammation were analyzed in relation to prostate cancer risk. Our preliminary data suggests that the possession of *IL1R2* rs11886877 locus modifies prostate cancer susceptibility among individuals with African ancestry in the U.S. However, the association of the *IL1R2* variant with prostate risk did not remain significant after adjust for multiple hypothesis testing. Future studies with ample statistical power to accommodate adjustment for multiple comparisons bias, will enable us to evaluate the impact of the *IL1R2* variant or a combination of inflammatory cytokine SNPs in relation to prostate cancer risk, tumor grade, biochemical or disease recurrence, and mortality. These studies will lead to the identification of genetic markers that modify the susceptibility of individuals.

## Abbreviations

SNP: Single nucleotide polymorphism; LR: Logistic regression.

## Competing interests

The authors declare that they have no competing interests.

## Authors’ contributions

LRK and KSK: conceptualized the project. LRK, KSK, CR, MJ, NM, MT, SM: participated in the study design. DZJ, LRK: composed the manuscript. DZJ, LRK, CR, REF: revised subsequent manuscript drafts. DZJ, NCK: quality control and statistical analysis. LRK: supervised quality control analysis, data-management and statistical analysis. LRK, DZJ, CR, KSK: interpreted the data, gave important intellectual input toward the introduction, results and/or discussion. All co-authors: read and edited the manuscript drafts as well as gave final approval of the final manuscript draft.
